# Supplementary material for: Expression of the microRNA-200 Family, microRNA-205, and Markers of Epithelial–Mesenchymal Transition as Predictors for Endoscopic Submucosal Dissection over Esophagectomy in Esophageal Adenocarcinoma: A Single-Center Experience
Source: Cells. 2020 Feb 20;9(2):486. doi: 10.3390/cells9020486 (PMC7072807; doi:10.3390/cells9020486)
Supplement: Supplementary file 1 [file cells-09-00486-s001.pdf]

Supplementary Information

Supplementary Table 1. Extended patient characteristics.

ID	L	V	Pn	R-Status	Tumor content (min-max, %)[b]
**endoscopic resection (ESD)**					
E1	0	0	0	0	5-10%
E2	0	0	0	0	10-15%
E3	0	0	0	0	15-20%
E4	0	0	0	0	5-10%
E5	0	0	0	0	40-50%
E6	0	0	0	0	10-15%
E7	0	0	0	0	50-60%
E8	0	0	0	0	5-10%
E9	0	0	0	0	20-30%
E10	0	0	0	0	5-10%
**endoscopic resection (ESD)**					
R1	1	0	1	1	60-70%
R2	1	1	0	1	20-30%
R3	1	1	0	0	30-40%
R4	1	0	0	1	30-40%
R5	1	0	0	0	10-20%
R6	1	0	0	0	30-40%
R7	1	0	1	1	80-90%
R8	1	0	1	0	80-90%
R9	1	0	0	0	5-10%
R10	1	1	1	0	50-60%

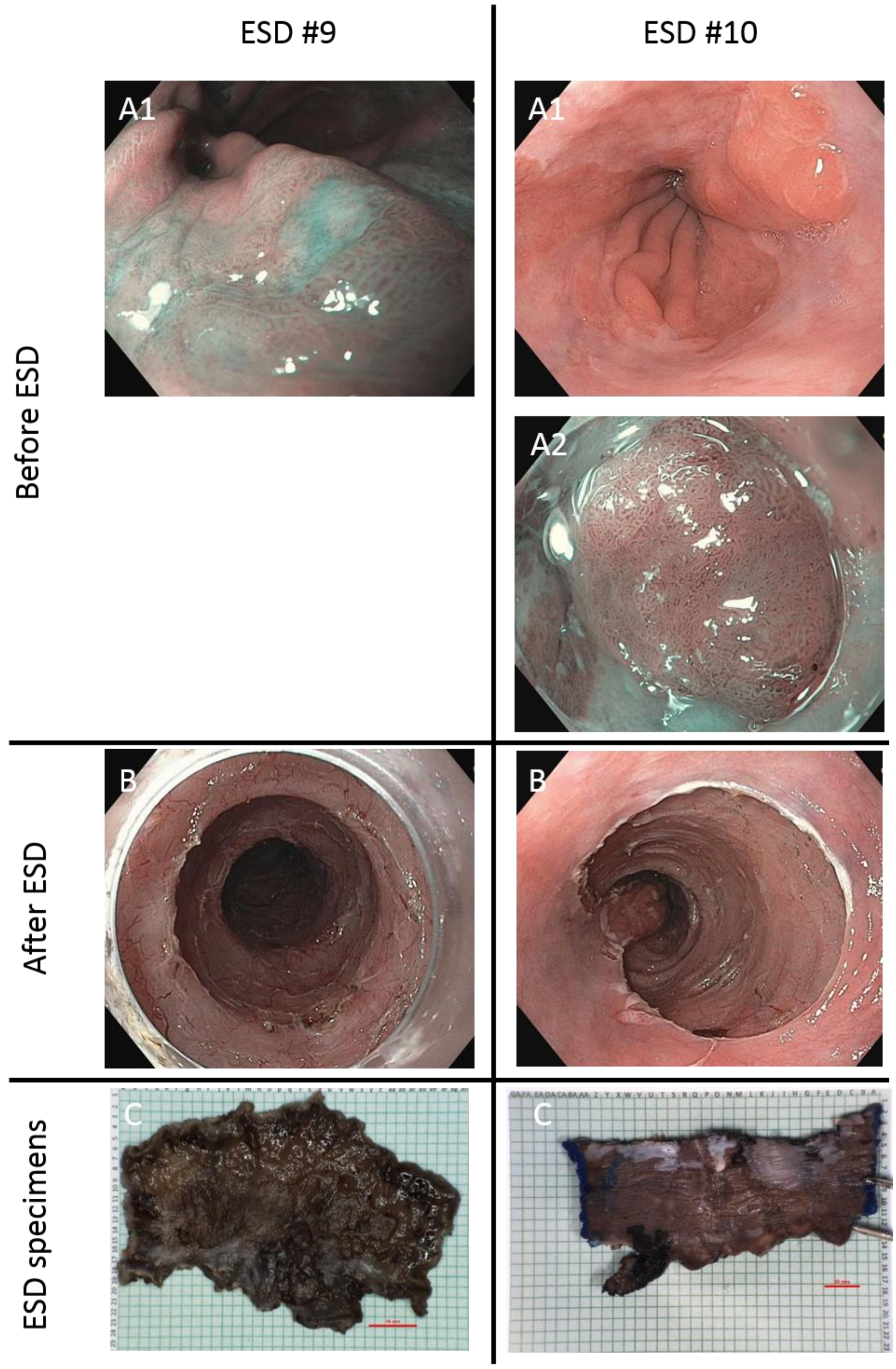

Supplementary Figure 1. Exemplary images of typical endoscopic aspects of esophogeal adenocarcinoma before and after endoscopic submucusal dissection (ESD, A and B) and corresponding ESD specimens (C). Shown are images of the immunohistochemical and molecular investigated ESD-cases #9 and #10 (E9 and E10). The additional applied narrow band imaging with near-focus mode (#9-A1 and #10-A2) highlight the irregular mucosal and pit pattern of the early esophogeal adenocarcinoma. The post-ESD images indicate the extension (in ESD-case #10 with complete circumferential due to extensive Barrett's mucosa) and clear margins of the endoscopic resected tumor specimens.
